# Oxidative Stress-Induced Unscheduled CDK1–Cyclin B1 Activity Impairs ER–Mitochondria-Mediated Bioenergetic Metabolism

**DOI:** 10.3390/cells10061280

**Published:** 2021-05-21

**Authors:** Jan-Gowth Chang, Ni Tien, Yi-Chih Chang, Meng-Liang Lin, Shih-Shun Chen

**Affiliations:** 1 Department of Laboratory Medicine, China Medical University Hospital, Taichung 404394, Taiwan; d6781@mail.cmuh.org.tw (J.-G.C.); t6719@mail.cmuh.org.tw (N.T.); 2 Department of Medical Laboratory Science and Biotechnology, College of Medical and Health Science, Asia University, Taichung 41354, Taiwan; yichih@asia.edu.tw; 3 Department of Medical Laboratory Science and Biotechnology, China Medical University, Taichung 404394, Taiwan; 4 Department of Medical Laboratory Science and Biotechnology, Central Taiwan University of Science and Technology, Taichung 406053, Taiwan

**Keywords:** apigenin, ASM, BCL-2/BCL-x_L_, cyclin B1–CDK1, ER–mitochondria, lipid raft, oxidative stress

## Abstract

Targeting the activities of endoplasmic reticulum (ER)–mitochondrial-dependent metabolic reprogramming is considered one of the most promising strategies for cancer treatment. Here, we present biochemical subcellular fractionation, coimmunoprecipitation, gene manipulation, and pharmacologic evidence that induction of mitochondria-localized phospho (p)-cyclin dependent kinase 1 (CDK1) (Thr 161)–cyclin B1 complexes by apigenin in nasopharyngeal carcinoma (NPC) cells impairs the ER–mitochondrial bioenergetics and redox regulation of calcium (Ca^++^) homeostasis through suppressing the B cell lymphoma 2 (BCL-2)/BCL-2/B-cell lymphoma-extra large (BCL-x_L_)-modulated anti-apoptotic and metabolic functions. Using a specific inducer, inhibitor, or short hairpin RNA for acid sphingomyelinase (ASM) demonstrated that enhanced lipid raft-associated ASM activity confers alteration of the lipid composition of lipid raft membranes, which leads to perturbation of protein trafficking, and induces formation of p110α free p85α–unphosphorylated phosphatase and tensin homolog deleted from chromosome 10 complexes in the lipid raft membranes, causing disruption of phosphatidylinositol 3-kinase (PI3K)−protein kinase B (Akt)−GTP-ras-related C3 botulinum toxin substrate 1 (Rac1)-mediated signaling, thus triggering the p-CDK1 (Thr 161))–cyclin B1-mediated BCL-2 (Thr 69/Ser 87)/BCL-x_L_ (Ser 62) phosphorylation and accompanying impairment of ER–mitochondria-regulated bioenergetic, redox, and Ca^++^ homeostasis. Inhibition of apigenin-induced reactive oxygen species (ROS) generation by a ROS scavenger N-acetyl-L-cysteine blocked the lipid raft membrane localization and activation of ASM and formation of ceramide-enriched lipid raft membranes, returned PI3K−Akt−GTP-Rac1-modulated CDK1–cyclin B1 activity, and subsequently restored the BCL-2/BCL-x_L_-regulated ER–mitochondrial bioenergetic activity. Thus, this study reveals a novel molecular mechanism of the pro-apoptotic activity of ASM controlled by oxidative stress to modulate the ER–mitochondrial bioenergetic metabolism, as well as suggests the disruption of CDK1–cyclin B1-mediated BCL-2/BCL-x_L_ oncogenic activity by triggering oxidative stress−ASM-induced PI3K−Akt−GTP-Rac1 inactivation as a therapeutic approach for NPC.

## 1. Introduction

Cholesterol- and sphingolipid-enriched membrane microdomains (lipid rafts) are highly dynamic in the cancer cells, which modulate the lateral compartmentalization of receptors and effector molecules at the cell surface to form a sorting platform essential for oncogenic signaling transduction [1,2]. Alterations in the bioenergetic metabolism and dynamics of organelles are emerging as critical event of carcinogenesis [3]. Two well-organized membrane-bound organelles, the endoplasmic reticulum (ER) and mitochondrion are now considered to act as the central organelles in the maintenance of metabolic reprogramming for increasing levels of the energy and lipid rafts in the cancer cells [1,3]. The regulatory molecules commanding the ER–mitochondrial functions are considered to be the B cell lymphoma 2 (BCL-2) family proteins, which can be identified as either pro-apoptotic or anti-apoptotic proteins [4]. The anti-apoptotic BCL-2/B-cell lymphoma-extra large (BCL-x_L_) and pro-apoptotic Bcl-2-associated x protein (BAX)/Bcl-2 antagonist killer 1 (BAK) proteins have a hydrophobic transmembrane anchoring domain at the carboxyl-terminus end, allowing they to reside on the membrane of ER and mitochondrion, conferring negative and positive regulation function in cell metabolism and survival [5,6]. In response to apoptotic stimuli, caused by either cellular or environmental stress, BAX and BAK change their conformations to form oligomers that associate not only with the mitochondrial membrane, but also with the ER membrane [7,8]. ER-targeted BAX/BAK oligomer causes the depletion of ER calcium (Ca^++^) and induces caspase-12 cleavage, whereas targeting of oligomeric BAX/BAK to mitochondria selectively induces the release of cytochrome *c* (Cyt *c*) from mitochondria and poly (ADP-ribose) polymerase (PARP) cleavage [5,7,9,10]. BCL-2 prevents oligomeric BAX/BAK-induced mitochondrial outer membrane permeabilization by direct binding to BAX and BAK [11]. The anti-apoptotic activity of BCL-2/BCL-x_L_ can be blocked by active cyclin dependent kinase 1 (CDK1)–cyclin B1 [12] through a mechanism that phosphorylates BCL-2 on threonine (Thr) 69 and serine (Ser) 87 [13] and catalyzes Ser 62 phosphorylation of BCL-x_L_ [12,14]. Cytoplasmic retention of CDK1–cyclin B1 complexes during damaged DNA-induced G_2_ arrest is thought to be involved in the binding of the 14-3-3σ [15]. An unscheduled CDK1–cyclin B1 activity in the mitochondria can also phosphorylate Ser residue 128 of Bcl-2-antagonist of cell death (BAD) causing a loss of interaction between BAD and 14-3-3 [16]. The resultant phosphorylated BAD (Ser 128) exists in the mitochondria to form heterodimers with BCL-2-realted proteins and/or BAX-like proteins, which are able to promote apoptosis [17]. As mentioned, active BAD can trigger conformational changes of BAX and BAK to promote homo-oligomerization of the proteins at the mitochondrial outer membrane upon apoptosis induction [18].

Under normal growth conditions, binding of the cyclin B1 to CDK1 is necessary for CDK1 activation and initiation of its phosphorylation at threonine residue 161 (Thr 161), thereby commit the mitochondrial metabolism for adaptive cell cycle progression and resistance of cancer cells [19,20]. Thr 161 phosphorylation is critical for maintenance of CDK1 kinase activity during the stage of mitosis [19,21], which is negatively regulated by the cyclin-dependent kinase inhibitor p21 [22]. Although the binding of p21 to CDK1 and its stability are involved in the nuclear accumulation of inactive CDK1–cyclin B1 complexes and the induction of apoptosis [23,24,25], caspase-3-mediated p21 cleavage confers DNA damage-induced apoptosis of cancer cells [26,27]. Enhanced stability of p21 provides survival of cancer cells through phosphorylation of p21 (Ser 146) by protein kinase B (Akt) [28]. A decrease in Akt activity was found associated with induction of caspase-3-mediated p21 cleavage, sustained phospho (p)-CDK1 (Thr 161)–cyclin B1 complex formation in human pharyngeal squamous carcinoma cells [29]. Active Akt exerts its action on the regulation of glucose metabolism through the inhibition of the conformational change of BAX during activation [30]. Akt regulates proliferation, survival, metabolism, and metastasis of cancer cells by phosphorylating various effectors, and its activity is triggered by phosphatidylinositol 3-kinase (PI3K) [31,32] and negatively regulated by phosphatase and tensin homolog deleted from chromosome 10 (PTEN) [33]. The exact mechanism linking between PI3K–Akt signaling and CDK1–cyclin B1 to dictate ER–mitochondria metabolic function in cancer cell survival remains an open issue.

Over-expression of BCL-2 and related family proteins (BCL-x_L_ and MCL-1) confers anti-apoptotic response and contributes to the development of resistance to chemotherapy and radiation treatment for nasopharyngeal carcinoma (NPC) [34]. A plant-derived flavonoid 4′,5,7-trihydroxyflavone (apigenin) was shown to induce radiosensitization of NPC cells by stimulating cyclin B1–CDK1 activity [35]. Thus, this study was conducted to explore the mitochondrial sequestration of cyclin B1–CDK1 complexes by apigenin that affecting BCL-2/BCL-x_L_-regulated ER–mitochondria bioenergetics and metabolism is dictated by PI3K–Akt-mediated signaling pathway in NPC cells.

## 2. Materials and Methods

### 2.1. Cell Culture

Epstein-Barr virus-negative human NPC cell lines (NPC-TW 039 and NPC-TW 076) with a G→C mutation at codon 280 causing an arginine to threonine amino acid change were derived from a 64-year-old male and a 36-year-old female Chinese patient with keratinizing squamous cell carcinoma (WHO type I) [36], respectively. Both cell lines were obtained as previously described [37]. The cell lines were cultured in Dulbecco’s Modified Minimum Essential Medium (DMEM) (Gibco BRL. Grand Island, NY, USA) supplemented with 5% FBS and grown in 10 cm tissue culture dishes at 37 °C in a humidified incubator containing 5% CO_2_.

### 2.2. Chemicals, Reagents, and Plasmids

Bismaleimidohexane (BMH), Brij 98, 4′,5,7-trihydroxyflavone (apigenin), crystal violet, dantrolene, 1,2-dioleoyl-*sn*-glycero-3-phosphocholine (DOPC), (5Z)-5-Quinolin-6-ylmethylene-2-[(thiophen-2-ylmethyl)-amino]-thiazol-4-one, 5-(6-Quinolinylmethylene)-2-[(2-thienylmethyl)amino]-4(5H)-thiazolone (Ro-3306), glutathione agarose beads, imipramine, propidium iodide (PI), ruthenium red, Tris-HCl, Triton X-100, and 3-(4,5-dimethylthiazol-2-yl)-2,5-diphenyltetrazolium bromide (MTT) were obtained from Sigma-Aldrich (St. Louis, MO, USA). The purity of apigenin by HPLC analysis was >95.00%. The inhibitor of pan-caspase (Z-VAD-FMK) was purchased from Calbiochem (San Diego, CA, USA) and dissolved in dimethyl sulfoxide (DMSO). DMSO and potassium phosphate were purchased from Merck (Darmstadt, Germany). Lipofectamine 2000 was obtained from Invitrogen (Carlsbad, CA, USA). DMEM, FBS, penicillin-streptomycin, trypsin-EDTA, and glutamine were obtained from Gibco BRL (Grand Island, NY, USA). The biotinylated annexin V was obtained from Thermo Fisher Scientific (New York, NY, USA). N-acetyl-L-cysteine (NAC) was purchased from Calbiochem (San Diego, CA, USA). Centricon YM-100 was obtained from Millipore. Acid sphingomyelinase (ASM) Assay Kit (Fluorimetric) was obtained from Abcam (Cambridge, MA, USA). The Amplex Red cholesterol assay kit was purchased from Molecular Probe (Eugene, OR, USA). The PCR-amplified BCL-2 (T69A/S87A), BCL-XL (S62A), BCL-2 (T69E/S87E), or BCL-XL (S62D) DNA was cloned into the pRK5-FLAG vector. pGFP shRNA was obtained from Addgene (Cambridge, MA, USA). ASM shRNA phasmid, CDK1 siRNA, cyclin B1 siRNA, control siRNA, and western blotting luminol reagent were purchased from Santa Cruz Biotechnology (Santa Cruz, CA, USA). CDK1 siRNA, cyclin B1 siRNA, and control siRNA were dissolved in RNase-free water. The sense strand siRNA sequences were as follows: CDK1 siRNA, 5′-CCUAUGGAGUUGUGUAUAATT-3′; cyclin-B1 siRNA, 5’- GUCAGUGAACAACUGCAGG-3’.

### 2.3. Antibodies

Antibodies against caspase-3, caspase-9, and caspase-12 were obtained from Calbiochem (San Diego, CA, USA). Anti- p85α -Akt, -phospho (p)-Akt (Ser 473), BAX, BAK, BCL-2, BCL-_XL_, and cytochrome *c* (Cyt *c*) antibodies were purchased from BD PharMingen. Antibodies directed against acid sphingomyelinase (ASM), BAD, p-BAD (Ser 128), p-BCL-2 (Ser 87), Bid, calnexin, p-CDK1 (Thr 161), Cyt *c* oxidase subunit II (Cox-2), and MCL-1 were obtained from Abcam (Cambridge, MA, USA). Antibodies against CD55, CD71, nucleolin, p21, CDK1, CDK2, cyclin A, cyclin B1, cyclin D, cyclin E, p110α, poly (ADP-ribose) polymerase (PARP), and Rac1 were purchased from Santa Cruz Biotechnology. Anti-pan-cadherin, anti-PTEN, and anti-p-PTEN (Ser 380/Thr 382/Ser 385) antibodies were obtained from Thermo Fisher Scientific (New York, NY, USA). Antibodies against β-actin, p-BCL-2 (Thr 69), p-BCL-_XL_ (Ser 62), FLAG-epitope tag, and α-tubulin were obtained from Sigma-Aldrich. Horseradish peroxidase (HRP)-conjugated streptavidin was provided from Thermo Fisher Scientific (New York, NY, USA). HRP-conjugated anti-mouse, -goat, and -rabbit IgG secondary antibodies were purchased from Jackson ImmunoResearch Laboratory (West Grove, PA, USA).

### 2.4. Measurement of DNA Fragmentation

Histone-associated DNA fragments were assayed using the Cell Death Detection enzyme-linked immunosorbent assay (ELISA) kit (Roche Applied Science, Mannheim, Germany). For the vehicle controls, DMSO was diluted in culture medium to the same final concentration (0.01%, *v/v*) as apigenin. Briefly, vehicle-treated or apigenin-treated cells (5 × 10^4^) were incubated in hypertonic buffer for 30 min at room temperature. After centrifugation, the cell lysates were transferred into an anti-histone-coated microplate to bind histone-associated DNA fragments. The plates were washed after 1.5 h of incubation, and nonspecific binding sites were saturated with blocking buffer. The plates were then incubated with peroxidase-conjugated anti-DNA for 1.5 h at room temperature. To determine the amount of retained peroxidase, 2,2′-azino-di-(3-ethylbenzthiazoline-6-sulfonate) was added as a substrate, and a spectrophotometer (Thermo Labsystems Multiskan Spectrum, Franklin, MA, USA) was used to measure the absorbance at 405 nm.

### 2.5. Subcellular Fractionation

Subcellular fractionation was performed according to the protocol of previously described [36]. The vehicle-treated or apigenin-treated cells (3 × 10^6^) were washed twice with ice-cold PBS and scraped into a 200 mM sucrose solution containing 25 mM HEPES (pH 7.5), 10 mM KCl, 15 mM MgCl_2_, 1 mM EDTA, 1 mM EGTA, and 1 μg/mL aprotinin. The cells were disrupted by passage through a 26-gauge hypodermic needle 30 times and then centrifuged for 10 min in an Eppendorf microcentrifuge (5804R) at 750× *g* at 4 °C to get the nucleus (N) pellet. The supernatant was collected and then centrifuged for 20 min at 6000× *g* at 4 °C to form a new supernatant and pellet. The resulting pellet was saved as the mitochondrial (Mt) fraction, and the supernatant was further centrifuged at 100,000× *g* for 1 h at 4 °C. The new supernatant was saved as the cytosolic (Cs) fraction, and the pellet was reserved as the ER/microsomal (Ms) fraction. The resulting N, Mt, Ms, and Cs fractions were lysed in RIPA buffer (1% sodium deoxycholate, 0.1% SDS, 1% Triton X-100, 10 mM Tris-HCl [pH 8.0], and 0.14 M NaCl) for Western blot analysis. The purity of each subcellular fraction was confirmed by Western blotting using specific antibodies against the nuclear marker nucleolin, the mitochondrial marker Cox-2, and the ER marker calnexin.

### 2.6. Density Based Membrane Flotation Technique

Detergent-resistant membranes (DRMs) were prepared as previously described [36]. Briefly, vehicle-treated, apigenin-treated, or NAC-treated cells (3 × 10^6^) were washed twice in ice-cold PBS before being removed from dishes by scraping. Cells were then harvested by centrifugation, resuspended in 1 mL of hypotonic lysis buffer (10 mM Tris [pH 7.5], 10 mM KCl, 5 mM MgCl_2_) containing 0.5% Brij 98, incubated at 37 °C for 5 min, and ruptured by passage through a 25-gauge hypodermic needle 20 times. Unbroken cells and nuclei were removed by centrifugation at 1000*× g* for 5 min in a microcentrifuge at 4 °C. The crude homogenates were kept on ice for an additional 5 min, mixed with 3 mL of 72% sucrose, and overlaid with 4 mL of 55% sucrose and 1.5 mL of 10% sucrose; all sucrose solutions were dissolved in low-salt buffer (50 mM Tris-HCl [pH 7.5], 25 mM KCl, 5 mM MgCl_2_). Samples were centrifuged for 14 h in a Beckman SW41 rotor at 38,000 rpm and 4 °C. Fractions were collected from the top of the gradient in 1-mL increments and concentrated to approximately 100 μL by passage through a 50-kDa Centricon filter.

### 2.7. Western Blot Analysis and Co-Immunoprecipitation

The vehicle-treated, apigenin-treated, NAC-treated, Ro-3306-treated, or CDK1/cyclin B1 siRNA-transfected cells (2.5 × 10^5^) were lysed and subjected to Western blotting as described previously [36]. Densitometric measurements of the bands in the Western blot analysis were performed using the computed densitometry and ImageQuant software (Molecular Dynamics, Sunnyvale, CA, USA). For the co-immunoprecipitation assays, cellular extracts were immunoprecipitated with anti-CDK1, anti-cyclin B1, anti-p85α, anti-p110α, anti-PTEN antibodies, or with normal control IgG, and then incubated with protein A agarose beads as previously described [36]. After incubation at 4 °C for 2 h, the immune complexes were analyzed by 10% SDS and immunoblotting with anti-cyclin B1, CDK1, p-CDK1 (Thr 161), anti-p85α, anti-110α, anti-Rac1, p-PTEN (Ser 380/Thr 382/Ser 385), and anti-PTEN antibodies.

### 2.8. Cell Surface Biotinylation

This assay was performed as previously described [38]. Briefly, vehicle-treated, apigenin-treated, Z-VAD-FMK-treated, or apigenin and Z-VAD-FMK-treated cells (2.5 × 10^5^) were washed twice in ice-cold PBS and incubated with 0.5 mg/mL of biotinylated annexin V for 30 min at 4 °C. Biotinylated cells were washed twice in ice-cold PBS and treated with 50 mM NH_4_Cl for 10 min at 4 °C to stop the biotinylation reaction. Avidin-agarose beads (Pierce, Rockford, IL, USA) were then added to the biotinylated cells, and the mixture was incubated with gentle rocking at 4 °C for 16 h. The beads were pelleted and washed three times with 500 μL of ice-cold PBS. Bound proteins were mixed with 1 × SDS sample buffer and incubated for 5 min at 100 °C. The proteins were then separated by 10% SDS-PAGE and immunoblotted with HRP-conjugated streptavidin.

### 2.9. Measurement of Cytosolic Ca^++^

The Ca^++^ level was determined by measuring the retention of Indo-1/AM. Briefly, the vehicle-treated, apigenin-treated, dantrolene-treated, apigenin and dantrolene-treated, ruthenium red-treated, apigenin and ruthenium red-treated, NAC-treated, or apigenin and NAC-treated cells (5 × 10^4^) were incubated with 3 μg/mL Indo-1/AM for 30 min at 37 °C. The cells were then pelleted by centrifugation at 160× *g*. The pellets were resuspended and washed twice with PBS. The level of Ca^++^ was evaluated as previously described [37].

### 2.10. Detection of Reactive Oxygen Species (ROS)

Briefly, the vehicle-treated, apigenin-treated, dantrolene-treated, apigenin and dantrolene-treated, ruthenium red-treated, apigenin and ruthenium red-treated, NAC-treated, or apigenin and NAC-treated cells (5 × 10^4^) were then resuspended in 500 μL of 2,7-dichlorodihydrofluorescein diacetate (10 μM) and incubated for 30 min at 37 °C. The level of ROS was determined using a FACSCount flow cytometer.

### 2.11. Plasmid and siRNA Transfection

Cells (5 × 10^4^) in 2-well plate were transfected with the FLAG-BCL-2 (T69A/S87A), FLAG-BCL-x_L_ (S62A), FLAG-BCL-2 (T69E/S87E), or FLAG- BCL-x_L_ (S62D), PTEN shRNA, or empty vector expression plasmid or with CDK1 siRNA, cyclin B1 siRNA, or control siRNA using Lipofectamine 2000. At 12 h after transfection, cells were treated with vehicle or apigenin for 36 h. The expression of FLAG-BCL-2 (T69A/S87A), FLAG-BCL-x_L_ (S62A), FLAG-BCL-2 (T69E/S87E), FLAG- BCL-x_L_ (S62D), PTEN, CDK1, and cyclin B1 in transfected cells was assessed by western blotting using antibodies specific to FLAG, BCL-2, BCL-x_L_, PTEN, CDK1, and cyclin B1.

### 2.12. Rac1 Activation Assay

The vehicle-treated or apigenin-treated cells (2.5 × 10^5^) or caveolin-1 and CD55-riched DRM fractions prepared from vehicle-treated or apigenin-treated cells (3 × 10^6^) were lysed by incubation with Rac1 lysis buffer (50 mM Tris-HCl (pH 7.4), 100 mM NaCl, 1 mM MgCl_2_, 20 mM β-glycerophosphate (pH 7.5), 1% NP-40, 10% glycerol, 10 mM NaF, 2 mM Na_3_VO_4_, 5 mM dithiothreitol, 0.5 mM phenylmethylsulfonyl fluoride, 1 μg/mL leupeptin, and 1 μg/mL pepstatin) for 15 min at 4 °C. The lysates were centrifuged at 14,000*× g* for 10 min in a microcentrifuge at 4 °C. The lysates and immunoprecipitated complexes were incubated with 40 μg of bacterially expressed glutathione-S-transferase (GST)-PAK-CD fusion protein pre-bound to glutathione agarose beads for 30 min at 4 °C. The beads were pelleted, washed with 500 μL of Rac1 lysis buffer, mixed with 1× SDS sample buffer (50 mM Tris-HCl (pH 6.8), 2% SDS, 0.1% bromophenol blue, 10% glycerol, and 100 mM dithiothreitol), and incubated for 5 min at 100 °C. The sample were then separated by *samples* *were* then *separated* 10% SDS-PAGE and immunoblotted with an antibody against Rac1.

### 2.13. Determination of Cholesterol, Sphingomyelin, and Ceramide

Fifty microliter of cell lysates, caveolin-1 and CD55-riched DRM fractions were extracted with 200 μL chloroform plus 200 μL methanol and then subjected to centrifugation at 12,000 rpm for 5 min. The bottom layer was collected and then *evaporated under vacuum* to a small pellet. The pellet was dissolved in 50 μL ethanol. Cholesterol levels were determined using an Amplex Red Cholesterol Assay Kit according to the manufacturer’s protocol. The amount of *s*phingomyelin and ceramide was quantified by thin-layer chromatography as previously described [36].

### 2.14. Measurement of Intracellular Glucose

The intracellular glucose of cells was measured using glucose assay kit (Abcam, Cambridge, MA, USA) according to the manufacturer’s instructions. Briefly, cells were plated in 24-well plates at the density of 2 × 10^4^ cells per well to allow for attachment overnight. The cells were grown to ≈60% confluence and treated with vehicle or apigenin for the indicated periods. At the end of the incubation, cell lysates of 1 μL were added to 96-well plates and the volume was adjusted to 50 μL/well with Lactate Assay Buffer before addition of 50 μL Glucose Reaction Mix (composed of 46 μL Glucose Assay Buffer, 1 μL Glucose Enzyme Mix, and 1 μL Glucose Probe) to each well and incubation at room temperature in the dark for 10 min. The absorbance was determined in a microplate reader (EL340 Bio-TEK Instruments, Winooski, VT, USA) at 570 nm. Glucose concentration was derived from absorbance using a standard curve.

### 2.15. Measurement of Cellular ATP

Cellular ATP content was measured by the ATP-based CellTiter-Glo Luminescent Cell Viability kit (Promega, Madison, MI, USA) with modifications from the manufacturer’s protocol. Briefly, cells were plated in 24-well plates at 2 × 10^4^ cells per well to allow for attachment overnight. The cells were grown to ~60% confluence and treated with vehicle or apigenin for the indicated periods. At the end of the incubation, an equal volume of the single-one-step reagent provided by the kit was added to each well and rocked for 15 min at room temperature. Cellular ATP content was measured by a luminescent plate reader. An additional plate with the same setup was used for cell counting by hemocytometer to normalize the cell number for calculating ATP level.

### 2.16. Statistical Analysis

Statistical calculations of the data were performed using the unpaired Student’s *t*-test and one-way ANOVA. *p* < 0.05 was considered statistically significant.

## 3. Results

### 3.1. BCL-2 (Thr 69 and Ser 87)/BCL-x_L_ (Ser 62) Phosphorylation and BAX/BAK ER–Mitochondrial Oligomerization Associated with Apigenin-Induced ER–Mitochondrial Dysfunction of NPC Cells

**Figure 1:**
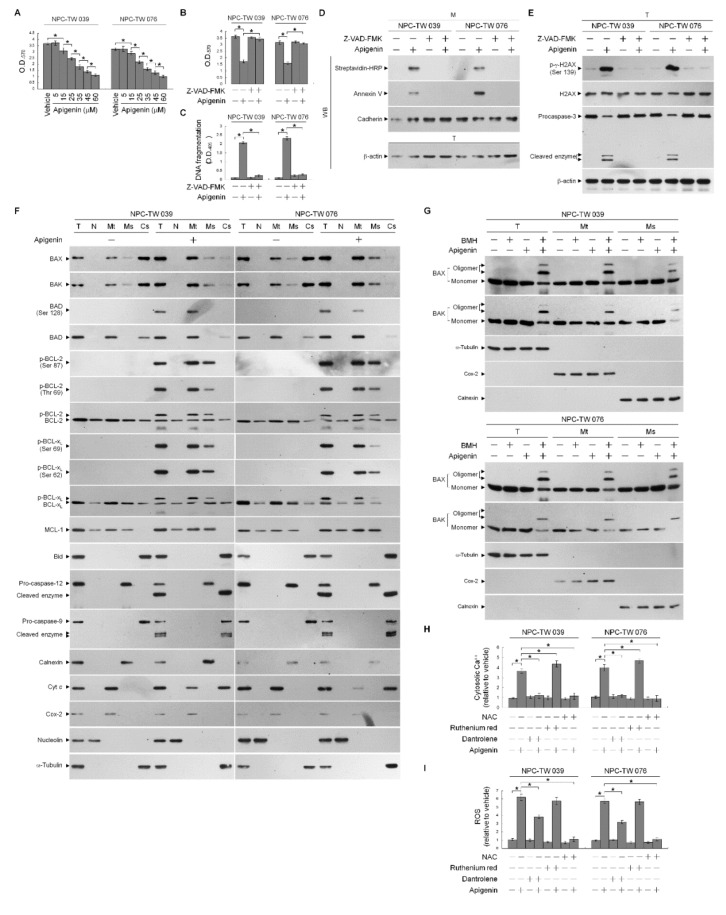
Apigenin-induced B cell lymphoma 2 (BCL-2) (Thr 69 and Ser 87)/BCL-2/B-cell lymphoma-extra large (BCL-x_L_) (Ser 62) phosphorylation and Bcl-2-associated x protein (BAX)/Bcl-2 antagonist killer 1 (BAK) endoplasmic reticulum (ER)–mitochondrial oligomerization associated with ER–mitochondria dysfunction and apoptotic death of nasopharyngeal carcinoma (NPC) cells. (**A**) The effect of apigenin on NPC cell growth. Cells were treated with vehicle (−) or the indicated concentrations of apigenin for 36 h. Cell growth was determined by the 3-(4,5-dimethylthiazol-2-yl)-2,5-diphenyltetrazolium bromide (MTT) assay. (**B**–**E**) The effects of apigenin on the induction of NPC cell growth inhibition and apoptosis. After a 36 h treatment with vehicle, apigenin (35 μM), Z-VAD-FMK (8 μM), or apigenin (35 μM) and Z-VAD-FMK (8 μM), DNA fragmentation was determined using a Cell Death Detection ELISA kit. Annexin V-biotinylated vehicle- or apigenin-treated cells were fractionated by subcellular fractionation centrifugation to isolate the plasma membrane (M) fraction. The level of the indicated proteins in the lysates of vehicle-, apigenin-, -Z-VAD-FMK, apigenin and Z-VAD-FMK co-treated total cell (T) and M fraction were determined by Western blot analysis using streptavidin-horseradish peroxidase (HRP) and specific antibody to Annexin V. Antibody against cadherin was used as internal controls for the plasma membrane. The levels of phosphorylated histone H2A.X (Ser 139) (p-γ-H2AX (Ser 139)), H2AX, and caspase-3 in the T were determined by Western blot analysis with specific antibodies. β-Actin was used as an internal control for sample loading. The effects of apigenin on the induction of BCL-2 (Thr 69 and Ser 87)/BCL-x_L_ (Ser 62) phosphorylation and Bax/Bak oligomerization in the ER and mitochondria of NPC cells (**F**) Cells were treated with apigenin for 36 h. Subcellular nuclear (N), mitochondrial (Mt), ER/microsomal (Ms), and cytosolic (Cs) fractions were separated by differential centrifugation. The levels of the indicated proteins in the lysates of the untreated or treated N, Mt, Ms, and Cs fractions were determined by Western blot analysis using specific antibodies. Antibodies against nucleolin, Cox-2, calnexin, and α-tubulin were used as internal controls for the nucleus, mitochondria, ER, and cytosol, respectively. (**G**) Cells were harvested 36 h after treatment with vehicle or apigenin, and cell pellets were resuspended in hypotonic buffer. Crude homogenates were incubated with 5 mM bismaleimidohexane (BMH) in phosphate buffered saline (PBS) for 30 min at room temperature and then subjected to subcellular fractionation to obtain the Mt and Ms fractions. In total, 20 μg of total protein from the recovered fractions was analyzed by 10% sodium dodecyl sulfate polyacrylamide gel electrophoresis (SDS-PAGE) and probed with specific antibodies, as indicated. Cox-2, calnexin, and α-tubulin were used as internal controls for the mitochondria, ER, and cytosol, respectively. (**H**,**I**) The effects of apigenin on the generation of cytosolic calcium (Ca^++^) and reactive oxygen species (ROS). Cells were treated with vehicle, apigenin (35 μM), dantrolene (25 μM), apigenin (35 μM), and dantrolene (25 μM), ruthenium red (1 μM), apigenin (35 μM), and ruthenium red (1 μM), N-acetyl-L-cysteine (NAC) (100 μM), or apigenin (35 μM) and NAC (100 μM) for 36 h, and the cytosolic level of Ca^++^ and ROS were monitored by measuring the increased fluorescence of Indo-1 and 2,7-dichlorodihydrofluorescein by flow cytometry. The values are presented as the means ± standard error of three independent experiments. * *p* < 0.05: significantly different from vehicle- or apigenin-treated cells.

In cells exposed to concentration ranging from 5 to 60 μM of apigenin for 36 h, apigenin effectively showed dose-dependent growth inhibition of nasopharyngeal carcinoma NPC-TW 039 and NPC-TW 076 cells, with half-maximal inhibitory concentration (IC_50_) value of 35 μM (Figure 1A). NPC cells through apoptosis display DNA fragmentation, Annexin-V binding, DNA double-strand break marker histone H2A.X (Ser 139) phosphorylation, and procaspase-3 cleavage when treated with an IC_50_ of 35 μM apigenin (Figure 1C–E). Apigenin-induced growth-suppressing and apoptotic effects were completely inhibited by a broad spectrum caspase inhibitor Z-VAD-FMK (Figure 1B–E). Thus, 35 μM concentration of apigenin was used to treat NPC cells in all subsequent experiments. Although previous studies have shown that alteration of the ER and mitochondria function involved in the apoptotic process of apigenin-treated cancer cells [39,40], the underlying mechanism has remained unclear. Since the BCL-2 proteins are key regulators of the maintenance of ER–mitochondria homeostasis to encourage evasion of cancer cell apoptosis [41]. Compartmentalization of the ER and mitochondrial membrane of BCL-2 proteins is critical for cell fate determination [4], we then analyzed the subcellular localization of BCL-2 family proteins in NPC cells. Subcellular fractionation was performed to isolate the crude nucleus (N), mitochondrial (Mt), ER/microsomal (Ms), and cytosolic (Cs) fractions. The N, mitochondria, ER, and cytoplasm apparatus were separated under the condition, as evidenced by the fact that antibodies detected nuclear marker nucleolin, Mt marker cytochrome (Cyt) *c* oxidase subunit II (Cox-2), ER marker calnexin, and cytosol marker α-tubulin in the N, Mt, Ms, and Cs fractions, respectively. Western blot analysis of the proteins in isolated fractions of vehicle-treated cells revealed that both BAX and BAK were found predominantly in the mitochondria and cytosol, they were also detected in a small amount in the ER, but did not show association with the nucleus, consistently with previous studies [42,43]. BCL-2 and BCL-x_L_ resided mostly in the mitochondria and ER fractions and partly located in the nucleus and cytosol. MCL-1 showed mainly subcellular localization to mitochondria and secondly distributed in the ER and nucleus, without any cytosolic presence. Exposure of NPC cells to apigenin reduced the levels of BAX and BAK in the cytosol accompanied with increases of BAX and BAK in the mitochondria and ER. The increased amount of BAD in the mitochondria was coincided with an induced presence of phosphorylated BAD (Ser 128) with mitochondria after apigenin treatment. Bid, a pro-apoptotic protein of the BCL-2 family, was present in the cytosol; however, its active truncated form (tBid), a known inducer of BAX/BAK mitochondrial translocation and oligomerization [44,45,46], was not detected in the mitochondria, and cleavage of cytosolic Bid was not found in apigenin-treated cells. Formation of p-BCL-2 (Thr 69 and Ser 87) and p-BCL-x_L_ (Ser 62) on the mitochondria and ER was observed upon treatment with apigenin. The cleaved active form of caspase-9 was induced and resided in the cytosol when cells treated with apigenin. Cytosolic localization of Cyt *c* was also found in apigenin-treated cells. The cleavage of ER-associated pro-caspase-12, resulting in the cytosolic localization of activated caspase-12, was induced by apigenin (Figure 1F). Given the potential effects of oligomeric BAX and BAK on the induction of mitochondria- and ER-initiated apoptosis signal [5,7,10], a sulfhydryl-reactive agent BMH was utilized to crosslink the oligomerized proteins in treated cells [7,43], followed by isolation of Mt and Ms fractions. By using COX-2 as a marker for the mitochondria and calnexin as a marker for the ER, Figure 1G showed that apigenin treatment resulted in induced the oligomerization of BAX and BAK in both mitochondria and ER that was represented by the presence of slowly immobilized bands recognized by BAX or BAK antibody. To investigate whether apigenin treatment resulted in Ca^++^ oscillation, the level of Ca^++^ in the cytosol was determined by flow cytometry. Indeed, cytosolic [Ca^++^] was increased in cells after treatment with apigenin, and this elevated level was completely inhibited by dantrolene, an inhibitor of Ca^++^ channel proteins, inhibiting the release of Ca^++^ from the ER [47,48]. Moreover, the dantrolene-induced increased the level of cytosolic Ca^++^ was attenuated by a ROS scavenger N-acetyl-L-cysteine (NAC). The cytosolic Ca^++^ concentrations was more raised than apigenin-treated cells by the co-addition of ruthenium red as an inhibitor of mitochondrial Ca^++^ uptake (Figure 1H) [49], indicating that the increase in mitochondrial Ca^++^ uptake was due to release from the ER. Apigenin-induced increase in reactive oxygen species (ROS) level was partially inhibited by dantrolene, but was completely suppressed by NAC (Figure 1I); however, ROS induction was not significantly altered by ruthenium red. These findings suggest that the phosphorylation of BCL-2 (Thr 69 and Ser 87)/BCL-x_L_ (Ser 62) and the targeted oligomerization of BAX and BAK on the mitochondria and ER caused by apigenin may result in cell apoptosis through ER–mitochondria dysfunction.

### 3.2. Apigenin-Induced ER–Mitochondria Metabolic Dysfunction Involved in Dysregulation of the Anti-Apoptotic Function of BCL-x_L_/BCL-2

**Figure 2:**
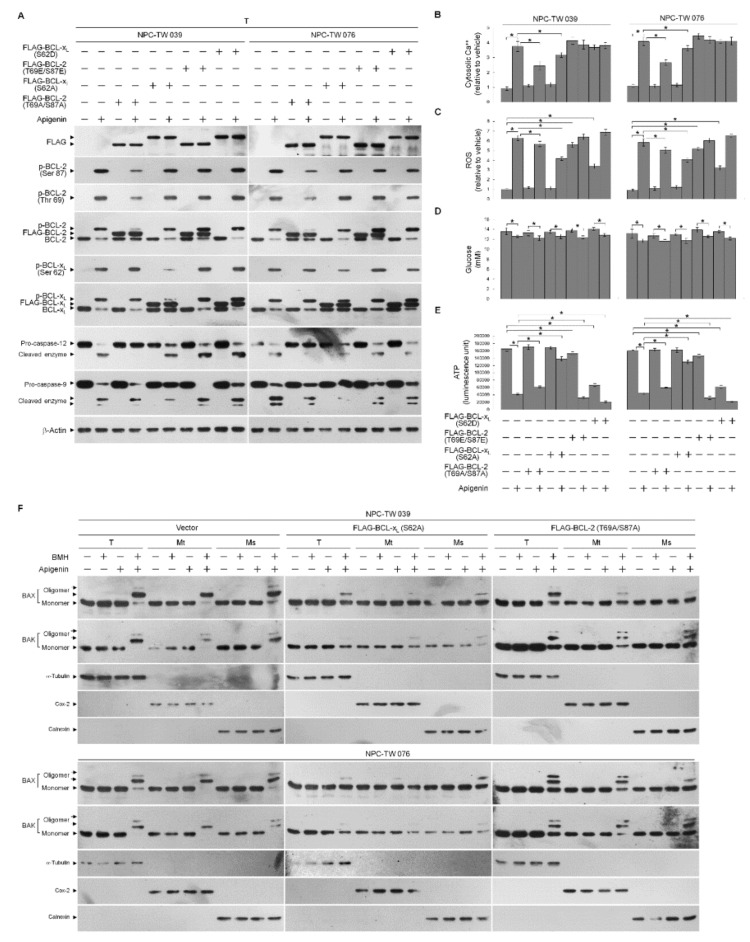
Apigenin-induced B cell lymphoma 2 (BCL-2) (Thr 69 and Ser 87)/BCL-2/B-cell lymphoma-extra large (BCL-x_L_) (Ser 62) phosphorylation contribute to endoplasmic reticulum (ER)–mitochondria oligomerization of Bcl-2-associated x protein (BAX)/Bcl-2 antagonist killer 1 (BAK) and affect generation of reactive oxygen species (ROS), calcium (Ca^++^), and adenosine triphosphate (ATP). (**A**–**E**) Effects of the ectopic expression of the FLAG-BCL-2 (T69A/S87A), FLAG-BCL-x_L_ (S62A), FLAG-BCL-2 (T69E/S87E), or FLAG- BCL-x_L_ (S62D) on the apigenin-induced of BAX/BAK oligomerization, ROS, cytosolic Ca^++^, and ATP levels. At 12 h after transfection with empty vector, FLAG-BCL-2 (T69A/S87A), FLAG-BCL-x_L_ (S62A), FLAG-BCL-2 (T69E/S87E), or FLAG- BCL-x_L_ (S62D), Nasopharyngeal carcinoma (NPC) cells were treated with vehicle (-) or apigenin (35 μM) for 36 h. The levels of the indicated proteins in the whole cell lysates of transfected cells were determined by Western blot analysis with specific or anti-FLAG antibodies. The generation of cytosolic level of Ca^++^ and ROS were monitored by measuring the increased fluorescence of Indo-1 and 2,7-dichlorodihydrofluorescein by flow cytometry. The values of glucose and ATP were analyzed using glucose assay and ATP-based CellTiter-Glo Luminescent Cell Viability kits, respectively. The values are presented as the means ± standard error of three independent experiments. * *p* < 0.05: significantly different from vehicle- or apigenin-treated cells. (**F**) At 12 h after transfection with empty vector, FLAG-BCL-2 (T69A/S87A) or FLAG-BCL-x_L_ (S62A), cells were treated with vehicle (-) or apigenin (35 μM) for 36 h. Cells were harvested and resuspended in hypotonic buffer. Crude homogenates were incubated with 5 mM bismaleimidohexane (BMH) in phosphate buffered saline (PBS) for 30 min at room temperature and then subjected to subcellular fractionation to obtain the mitochondrial (Mt) and ER/microsomal (Ms) fractions. In total, 20 μg of total protein from the recovered fractions was analyzed by 10% sodium dodecyl sulfate polyacrylamide gel electrophoresis (SDS-PAGE) and probed with specific antibodies, as indicated. Cox-2, calnexin, and α-tubulin were used as internal controls for the mitochondria, ER, and cytosol, respectively.

To further address an action between BCL-2/BCL-x_L_ phosphorylation and oligomeric states of BAX/BAK in dysregulation of ER–mitochondria metabolism, transiently transfected cells expressing FLAG epitope-tagged phosphodefective BCL-2 (T69A/S87A)/BCL-x_L_ (S62A) or phosphomimetic BCL-2 (T69E/S87E)/BCL-x_L_ (S62D) were performed and verified by Western blot analysis using specific antibodies against FLAG, BCL-2, or BCL-x_L_ (Figure 2A). Vehicle-treated cells ectopically expressing FLAG-BCL-2 (T69A/S87A) or FLAG-BCL-x_L_ (S62A) did not affect the levels of cellular Ca^++^, ROS, glucose, and ATP (Figure 2B–E). The induction of BAX/BAK oligomerization at the ER/mitochondria and the decrease of ATP level by apigenin were profoundly overcome by FLAG-BCL-x_L_ (S62A) expression, and these effects were partially inhibited by FLAG-BCL-2 (T69A/S87A) (Figure 2E,F). Overexpression of FLAG-BCL-2 (T69A/S87A) suppressed apigenin-stimulated increase in cytosolic Ca^++^; however, FLAG-BCL-x_L_ (S62A) overexpression had little effect on the suppression of apigenin-induced cytosolic Ca^++^ elevation (Figure 2B). The apigenin-stimulated a robust production of ROS was markedly attenuated by a forced overexpression of FLAG-BCL-x_L_ (S62A), whereas was slightly affected by FLAG-BCL-2 (T69A/S87A) overexpression (Figure 2C). As the meantime, apigenin-induced cleavage of ER-specific pro-caspase-12 was completely suppressed by FLAG-BCL-2 (T69A/S87A), but not by FLAG-BCL-x_L_ (S62A). Conversely, FLAG-BCL-x_L_ (S62A), but not FLAG-BCL-2 (T69A/S87A), was able to attenuate the induction of cleaved caspase-9 by apigenin (Figure 2A). Apigenin caused a slight decline in glucose that was not affected by a FLAG-BCL-x_L_ (S62A) or BCL-2 (T69A/S87A) overexpression (Figure 2D). Ectopic expression of BCL-x_L_ (S62D) in vehicle-treated cells apparently causes a decrease in production of ATP as well as an increase of ROS, whereas generation of ROS, and ATP only marginally affected by BCL-2 (T69E/S87E) overexpression. The intracellular glucose level did not exhibit any change in BCL-x_L_ (S62D)- or BCL-2 (T69E/S87E)-transfected cells (Figure 2D). BCL-x_L_ (S62D) or BCL-2 (T69E/S87E) overexpression was not significantly appeared increase of the inductive effects of apigenin on the levels of ROS, Ca^++^, cleaved caspase-9/-12, but enhanced the inhibitory effect of apigenin on the generation of and ATP (Figure 2A–C,E). Thus, apigenin-evoked BCL-x_L_ (Ser 62) and BCL-2 (Thr 69 and Ser 87) phosphorylation raised ER–mitochondrial oligomerization of BAX/BAK that leads to dysregulation of ER–mitochondrial communication, which in turn causes impairment of energy metabolism, redox, and Ca^++^ homeostasis.

### 3.3. Induction of Unscheduled P-CDK1 (Thr 161)–Cyclin B1 Complexes by Apigenin Confers Dysregulation of the ER/Mitochondria Bioenergetic and Metabolic Control of BCL-2/BCL-x_L_

**Figure 3:**
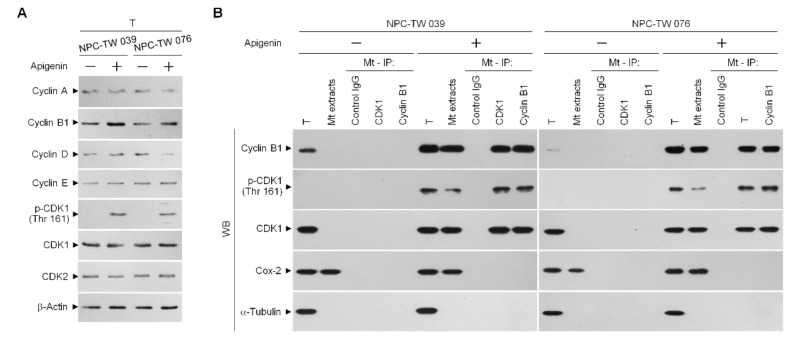
Induction of the formation of phospho (p)-cyclin dependent kinase 1 (CDK1) (Thr 161)–cyclin B1 complexes by apigenin. Nasopharyngeal carcinoma (NPC) cells were treated with vehicle (−) or apigenin (35 μM) for 36 h. (**A**) The levels of the indicated proteins in the total cell (T) lysates were determined using Western blot analysis with specific antibodies. β-Actin was used as an internal control for sample loading. (**B**) The antibody used for coimmunoprecipitation is indicated at the top. The proteins from the immunoprecipitated complexes were detected using Western blotting with specific antibodies. Normal IgG was used as a control for antibody specificity.

Active, phosphorylated CDK1 (Thr 161)–cyclin B1 complex has previously demonstrated its ability to inactivate anti-apoptotic action of BCL-x_L_ and BCL-2 by triggering their phosphorylation [12,13,14]. Incubation with apigenin increases the level of cyclin B1 and induces the phosphorylation of CDK1 (Thr 161) (Figure 3A). Using an antibody specific for CDK1, we performed coimmunoprecipitation experiments and Western blot analysis of the co-immunoprecipitates from the mitochondrial fractions of apigenin-treated cells showed that phospho (p)-CDK1 (Thr 161) formed a complex with cyclin B1. Reciprocal coimmunoprecipitation using an anti-cyclin B1 antibody followed by immunoblot analysis has evidenced a complex formation of p-CDK1 (Thr 161)–cyclin B1. In contrast, control immunoglobulin G (IgG) antibodies did not immunoprecipitate any specific protein that interacted with cyclin B1 or CDK1 protein (Figure 3B), confirming the specificity of the p-CDK1 (Thr 161)–cyclin B1 complexes in the mitochondria of apigenin-treated cells.

**Figure 4:**
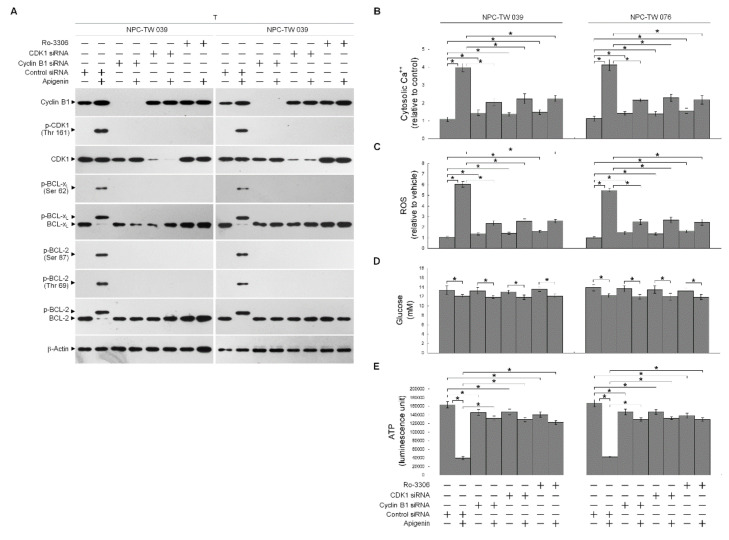
Apigenin-induced phospho (p)-cyclin dependent kinase 1 (CDK1) (Thr 161)–cyclin B1 activity involved in dysregulation of the endoplasmic reticulum (ER)/mitochondria bioenergetic metabolism. (**A**–**E**) At 12 h after transfection with control small interfering RNA (siRNA), cyclin B1 siRNA, or CDK1 siRNA, NPC cells were treated with vehicle (-), apigenin (35 μM), Ro-3306 (3 μM), or apigenin (35 μM), and Ro-3306 (3 μM) for 36 h. The levels of the indicated proteins in the whole cell lysates of transfected cells were determined by Western blot analysis with specific antibodies. The levels of cytosolic calcium (Ca^++^) and reactive oxygen species (ROS) were determined by measuring the increased fluorescence of Indo-1 and 2,7-dichlorodihydrofluorescein by flow cytometry. The values of glucose and adenosine triphosphate (ATP) were analyzed using glucose assay and ATP-based CellTiter-Glo Luminescent Cell Viability kits, respectively. The values are presented as the means ± standard error of three independent experiments. * *p* < 0.05: significantly different from vehicle- or apigenin-treated cells.

In order to verify the roles of elevated cyclin B1 level and CDK1 activity in the BCL-x_L_/BCL-2 phosphorylation, siRNA interference studies were performed. Western blot analysis indicated both the cyclin B1 and CDK1 siRNAs effective downregulation of cyclin B1 and CDK1 at protein level (Figure 4A). Downregulation of cyclin B1 or CDK1 with siRNA or suppression of CDK1 activity by a selective ATP-competitive inhibitor (Ro-3306) of CDK1 briefly elevated the cytosolic Ca^++^ and ROS levels and slightly decreased the ATP generation (Figure 4B,C,E). However, silencing of cyclin B1 or CDK1 expression in apigenin-treated cells by siRNA was restored un-phosphorylation status of BCL-x_L_/BCL-2 and the regulation of mitochondria glucose metabolism as efficiency of ATP generation (Figure 4A,B,D,E). Apigenin caused an increase in cytosolic Ca^++^ and ROS concentration, which was suppressed by cyclin B1 siRNA or CDK1 siRNA (Figure 4B,C). Consistent with knockdown results of CDK1 siRNA or cyclin B1 siRNA, pharmacological inhibition of CDK1 activity by a Ro-3306 also blocked the apigenin-induced phosphorylation of BCL-x_L_ (Ser 62) and BCL-2 (Thr 69 and Ser 87) (Figure 4A). Further, the suppressive effect of apigenin on the generation of ATP was overcome by Ro-3306 (Figure 4E). Moreover, like CDK1 siRNA, Ro-3306 co-treatment exhibited inhibitory effect on the induction of cytosolic Ca^++^ and ROS (Figure 4B,C). These results indicate that apigenin impaired bioenergetics metabolism through suppressing the regulation of the ER/mitochondria anti-apoptotic and energy metabolic control of BCL-2/BCL-x_L_, which was associated with the induction of active p-CDK1 (Thr 161)–cyclin B1 complexes.

### 3.4. Induction of Lipid Raft-Associated ASM-Mediated Ceramide Generation by Apigenin-Induced Oxidative Stress Impedes the Lipid Raft Membrane-Associated p85α−GTP−Rac1- Akt Signaling, Leading to the Sustained Formation of p-CDK1 (Thr 161)–Cyclin B1 Complexes

**Figure 5:**
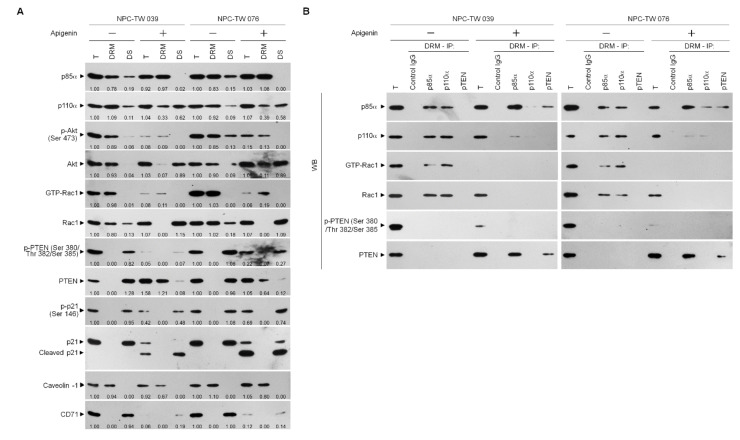
Apigenin induces p110α-free p85α–unphosphorylated phosphatase and tensin homolog deleted from chromosome 10 (PTEN) complex formation in the lipid raft membranes. (**A**) Nasopharyngeal carcinoma (NPC) cells were treated with vehicle (−) or apigenin (35 μM) for 36 h. The levels of the indicated proteins in the lysates of vehicle- or apigenin (35 μM)-treated total cell lysates (T), detergent-resistant membrane (DRM) and detergent-soluble (DS) fractions were determined by Western blot analysis using specific antibodies. The values above the figures represent relative density of the bands normalized to vehicle-treated proteins of T. (**B**) Coimmunoprecipitation of p85α, p110α, and PTEN was performed using the DRM fractions prepared from the cells treated as described above. The antibody used for coimmunoprecipitation is indicated at the top. The proteins from the immunoprecipitated complexes were detected using Western blotting with specific antibodies. Normal IgG was used as a control for antibody specificity.

The existence of previous evidences that attenuation of PI3K-Akt signaling primed the sustained presence of p-CDK1 (Thr 161)–cyclin B1 complexes by increasing the expression of cyclin B1 [29,50]. The absence of p21, a CDK1 inhibitor, causes a hyperactive CDK1–cyclin B1 [51]. Akt phosphorylates p21 at Ser 146 increasing it protein stability for action on the inhibition of CDK1 activity and enhances cancer cell survival [28]. The elevated caspase-3-mediated p21 cleavage caused by Akt inactivation, the prolonged p-CDK1 (Thr 161)–cyclin B1 complex formation [29]. Given the spatial compartmentalization of PI3K–Akt signaling components in the lipid raft membranes provides a platform hubs in cancer cell metabolism [52], we sought to determine whether a dysregulation of the lipid raft membrane targeting of PI3K–Akt signaling-regulated molecules occurred. One percent Brij 98 solubilization of apigenin-treated cell extracts was subjected to a sucrose density gradient centrifugation to isolate detergent-resistant membranes (DRMs), which are biochemically defined as the lipid rafts [2,53]. The DRM fractions were clarified by the presence of the lipid raft marker caveolin-1, and transferrin receptor CD71 was used to determine the detergent-soluble (DS) non-lipid raft fractions. In vehicle-treated cells, p85α, p110α, active Akt (Ser 473 phosphorylated Akt; p-Akt (Ser 473)), and active Rac1 (GTP-bound Rac1; GTP-Rac1) mostly localized to the DRMs, while only minority of p85α, p110α, p-Akt (Ser 473), and inactive Rac1 in DS fractions were detected. The phosphatase-inactive form of phospho-PTEN (p-PTEN) (Ser 380/Thr 382/Ser 385) was associated preferentially with the DR fractions, whereas no detection of the biologically active form of PTEN (unphosphorylated PTEN; PTEN) was presented in the DRMs. A decrease of the DRM localization of p110α and an increase of p110α in the DS fractions were observed after addition of apigenin. The expression pattern of caveolin-1 in the DRMs of apigenin-treated cells was slightly decreased as compared to that of vehicle-treated DRMs. The levels of p-Akt (Ser 473) and GTP-Rac1 associate with the DRMs were profoundly decreased by apigenin, and accompanied with increase of unphosphorylated Akt and Rac1 in the DR fractions. At the meantime, apigenin treatment causes of increased levels of p85α in the DRMs by recruiting the DS fraction p85α. An enhanced targeting of unphosphorylated PTEN localizing to the DRMS was observed, which was correlated with the decrease of p-PTEN (Ser 380/Thr 382/Ser 385) levels in the DS fractions. Inhibition of p21 (Ser 146) phosphorylation was found, and a cleaved form of p21 also detected in the apigenin-treated DS fractions (Figure 5A). These results suggest that recruitment of unphosphorylated PTEN to lipid rafts by apigenin may contribute to the suppression of PI3K–Rac1–Akt signaling, leading to the inactivation of cleavage of p21. Since the formation of p110α-free p85α-unphosphorylated PTEN complexes in the lipid raft members is crucial for PTEN-mediated suppression of the PI3K-dependent Akt activation [54], we examined the physical interaction between p85α, p110α, Rac1, or PTEN in the lipid raft membranes. In vehicle-treated cells, immunoprecipitation of p85α with specific antibody, followed by Western blot analysis of immunoprecipitates detected high levels of p110α and GTP-Rac1, but no detection of unphosphorylated PTEN in the DRMs. Conversely, an antibody against p110α was able to pull down substantial amount of p85α, p110α, and GTP-Rac1 but not of unphosphorylated PTEN. Further coimmunoprecipitation with PTEN antibody displayed the absence of the detection of unphosphorylated PTEN in the DRMs of vehicle-treated cells. Immunoprecipitated proteins of anti-p85α antibody obtained from the DRMs of apigenin-treated cells clearly included p85α:p110α:unphosphorylated PTEN. Immunoprecipitates obtained with anti-p110α antibody contained p85α:p110α without unphosphorylated PTEN and GTP-Rac1; however, reduced coimmunoprecipitation of p85α and p110α was found. Failure to co-immunoprecipitate GTP-Rac1 was observed with p85α or p110α antibody. Re-immunoprecipitation with anti-PTEN antibody revealed that a complex with p110α-free p85α and unphosphorylated PTEN was presented in the DRMs of apigenin-treated cells (Figure 5B), indicating that a complex formation of p110α-free p85α-unphosphorylated PTEN was induced in the lipid rafts of apigenin-treated cells.

**Figure 6:**
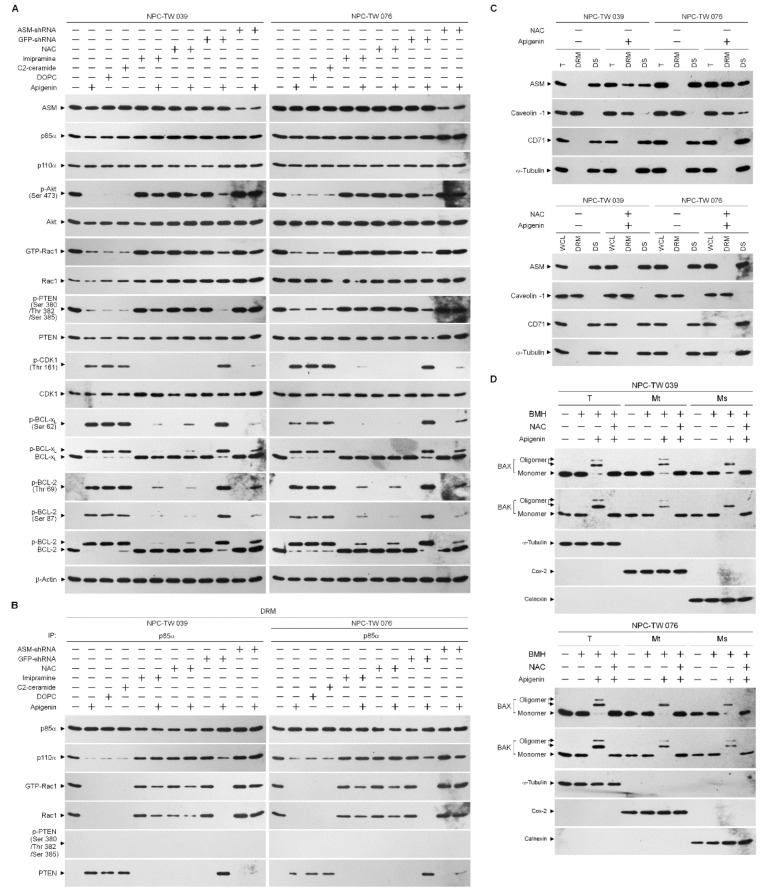

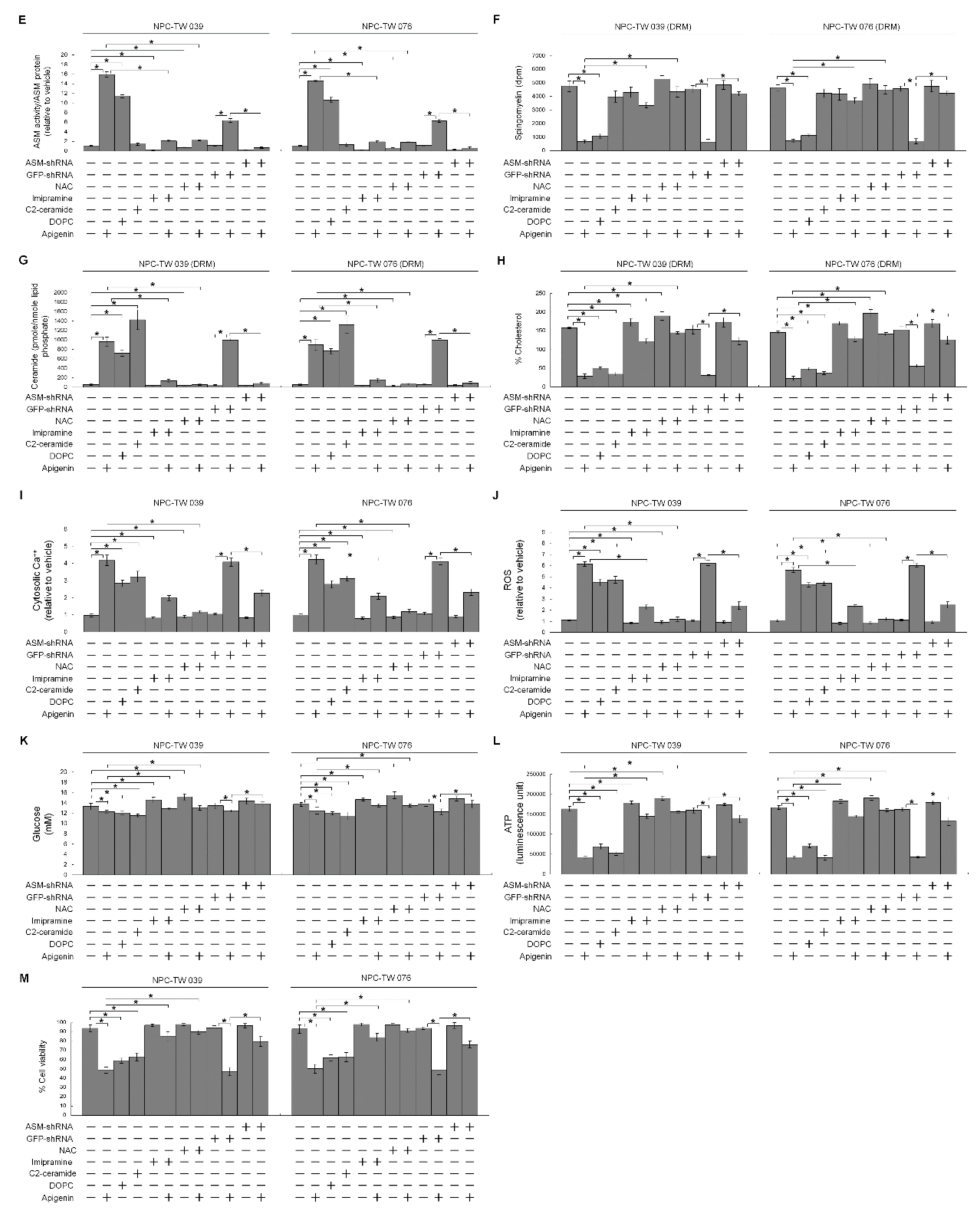
Apigenin-induced oxidative stress-dependent lipid raft-associated acid sphingomyelinase (ASM) activity causes dysregulation of the endoplasmic reticulum (ER)/mitochondria bioenergetic and lipid metabolism. (**A**,**B**,**E**–**M**) At 12 h after transfection with an empty vector, GFP shRNA or ASM shRNA cells were treated with either vehicle (−), apigenin (35 μM), 1,2-dioleoyl-*sn*-glycero-3-phosphocholine (DOPC) (15 nmole), C2-ceramide (6 μM), imipramine (5 μM), apigenin (35 μM) plus imipramine (5 μM), N-acetyl-L-cysteine (NAC) (100 μM), or apigenin (35 μM) and NAC (100 μM) for 36 h. The levels of the indicated proteins in the cell lysates were determined by Western blot analysis with specific antibodies. β-Actin was used as an internal control for sample loading. Detergent-resistant membranes (DRM) fractions were prepared by flotation on a sucrose density gradient. The antibodies used for co-immunoprecipitation are indicated at the top of the figure. The proteins in the immunoprecipitated complexes from the DRM were analyzed by Western blot using specific antibodies. ASM activities were determined by Acidic Sphingomyelinase Assay Kit. The lipids were extracted from the DRM fractions, and cholesterol, sphingomyelin, and ceramide were quantitated by Amplex Red Cholesterol Assay Kit and thin-layer chromatography, respectively. Ceramide concentrations were normalized to phospholipid phosphate. The generation of cytosolic level of calcium (Ca^++^) and reactive oxygen species (ROS) were monitored by measuring the increased fluorescence of Indo-1 and 2,7-dichlorodihydrofluorescein by flow cytometry. The values of glucose and adenosine triphosphate (ATP) were analyzed using glucose assay and ATP-based CellTiter-Glo Luminescent Cell Viability kits, respectively. Cell viability was determined by the flow cytometric analysis of PI uptake. The values are presented as the means ± standard error of three independent experiments. * *p* < 0.05: significantly different from vehicle- or apigenin-treated cells. (**C**) Cells were treated with vehicle (−), apigenin (35 μM), or apigenin (35 μM) and NAC (100 μM) for 36 h. DRM and detergent-soluble (DS) fractions were prepared by flotation on a sucrose density gradient. The levels of the indicated proteins in the lysates of vehicle- or apigenin-treated DRM and DS fractions were determined by Western blot analysis using specific antibodies. (**D**) Cells were treated with vehicle (−), apigenin (35 μM), or apigenin (35 μM) and NAC (100 μM). Cells were harvested 36 h after treatment with vehicle or apigenin, and cell pellets were resuspended in hypotonic buffer. Crude homogenates were incubated with 5 mM bismaleimidohexane (BMH) in PBS for 30 min at room temperature and then subjected to subcellular fractionation to obtain the mitochondrial (Mt) and ER/microsomal (Ms) fractions. In total, 20 μg of total protein from the recovered fractions was analyzed by 10% sodium dodecyl sulfate polyacrylamide gel electrophoresis (SDS-PAGE) and probed with specific antibodies, as indicated. Cox-2, calnexin, and α-tubulin were used as internal controls for the mitochondria, ER, and cytosol, respectively.

The results presented above show that apigenin-induced elevation of cytosolic Ca^++^ was blocked by a ROS scavenger NAC. Translocation of acid sphingomyelinase (ASM) from the lysosome to the lipid raft membranes drives the lipid raft recruitment and lipid phosphatase activity of PTEN that opposes PI3K-mediated Akt activation by dephosphorylating PIP_3_ into PIP_2_ [55,56]. Oxidative stress results from increased content of ROS that can trigger the plasma membrane translocation of Ca^++^-dependent ASM causing the formation of ceramide-enriched membrane platforms [57], we thought that lipid raft formation of p110α-free p85α–unphosphorylated PTEN complexes might be caused by an action of lipid raft membrane ASM-derived ceramide generation upon oxidative stress. To gain the evidence supporting this hypothesis, we treated cells with NAC or specific inducer, inhibitor, or shRNA for ASM and then performed a sucrose density-based membrane flotation to harvest the caveolin-1-enriched DRMs. Expression level of ASM remained unchanged, but its activity was increased after apigenin treatment (Figure 6A,E). Analysis of the protein contents of the DRMs of apigenin-treated cells showed that ASM was detected and co-fractioned with caveolin-1 but not with CD71, while the DS level of ASM was decreased (Figure 6C). Meanwhile, the levels of sphingomyelin and cholesterol were reduced, and concentration of ceramide was elevated in the DRMs of apigenin-treated cells (Figure 6F–H). Thus, the biochemical fractionation examination indicates that induced localization of ASM in the membrane lipid rafts was occurred by apigenin. The treatment of cells with 1,2-dioleoyl-*sn*-glycero-3-phosphocholine (DOPC), an inducer of ASM-mediated ceramide generation [58], raised ASM activity and DRM ceramide level, and caused p110α-free p85α–unphosphorylated PTEN complex formation (Figure 6B,E,G). DOPC addition also resulted in inhibition of the Akt (Ser 473)/PTEN (Ser 380/Thr 382/Ser 385) phosphorylation and Rac1 activation and reduced the levels of DRM sphingomyelin and cholesterol; moreover, enhanced induction of p-CDK1 (Thr 161), p-BCL-x_L_ (Ser 62), p-BCL-2 (Thr 69/Ser 87), cytosolic Ca^++^ and ROS and declined levels of glucose and ATP, and cell viability were found on treatment with DOPC (Figure 6A,F,H–M). Similar results of DOPC treatment were obtained with C2-ceramide, although ASM activity and DRM sphingomyelin were not altered by C2-ceramide (Figure 6A,B,E–M). As expected, ASM specific inhibitor imipramine was slightly elevating activation of PI3K–Rac1–Akt signal and levels of DRM cholesterol, PTEN (Ser 380/Thr 382/Ser 385) phosphorylation, glucose, and ATP as well as decreasing of Ca^++^ and ROS, but was not affecting DRM sphingomyelin and cell viability. The co-treatment of imipramine along with apigenin restored the cholesterol and sphingomyelin levels of DRMs, p85α−GTP-Rac1 complex formation, un-phosphorylation status of CDK1 and BCL-x_L_/BCL-2, levels of p-Akt, GTP-Rac1, p-PTEN (Ser 380/Thr 382/Ser 385), Ca^++^, ROS, glucose, ATP, and cell survival, and showed a substantial suppression of p85α–unphosphorylated PTEN formation in the DRMs (Figure 6A,B,E–M). Consistent findings in imipramine, knockdown of ASM in cells by shRNA was not affected cell viability and DRM sphingomyelin, but slightly increased the levels of p-Akt (Ser 473), p-PTEN (Ser 380/Thr 382/Ser 385), GTP-Rac1, glucose, and ATP as well as reduced levels of Ca^++^ and ROS, and countered from inductive and inhibitory effects of apigenin on the cells (Figure 6A,B,E–M). Significantly, blocking the increase of ROS generation by co-treatment of NAC with apigenin exhibited rescue effects on the suppression of p85α−GTP-Rac1 complex formation, Akt (Ser 473)/PTEN (Ser 380/Thr 382/Ser 385) phosphorylation, Rac1 activation, DRM cholesterol/sphingomyelin, glucose, ATP generation, and cell survival as well as displayed suppressive effect on the inhibition of the induction of CDK1 (Thr 161) phosphorylation, BCL-x_L_(Ser 62)/BCL-2 (Thr 69/Ser 87) phosphorylation, and oligomerization of ER−mitochondrial BAX/BAK, and as expected, p85α was not co-immunoprecipitated with PTEN. Moreover, apigenin-induced increase of ASM activity and cytosolic Ca^++^ was completely attenuated by co-addition with NAC (Figure 6A,B,D–M). Hence, it was indicated that apigenin triggers oxidative stress to induce the release of ER Ca^++^, which leads to membrane lipid raft trafficking of ASM to form ceramide-enriched lipid raft membranes and then induction of p110α-free p85α–unphosphorylated PTEN complexes, leading to disruption of p85α−GTP-Rac1−Akt-mediated signaling. The resultant inactive Akt causes impairment of CDK1−cyclinB1-BCL-2/BCL-_XL_-modulated ER–mitochondria-mediated bioenergetic and metabolic homeostasis.

## 4. Discussion

**Figure 7:**
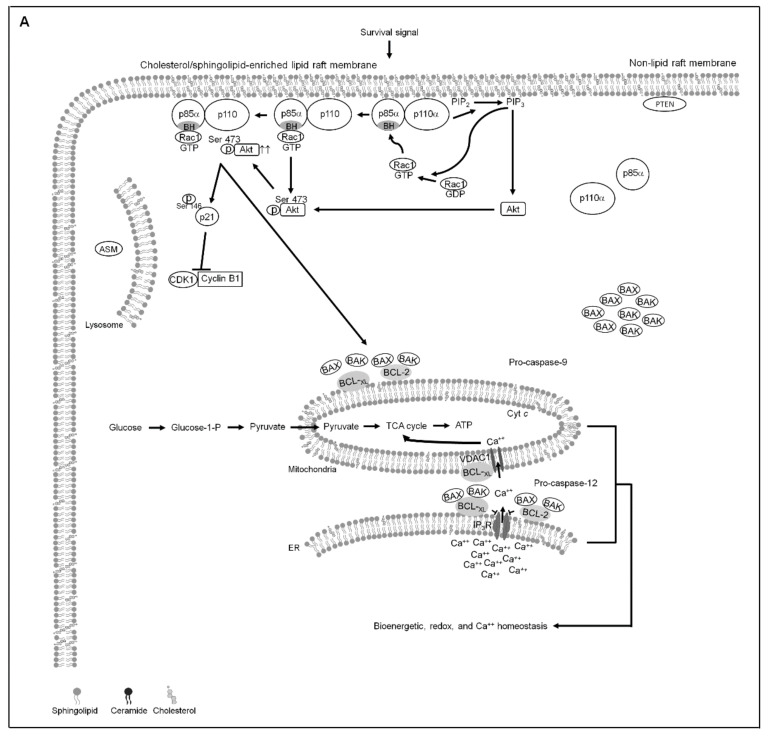

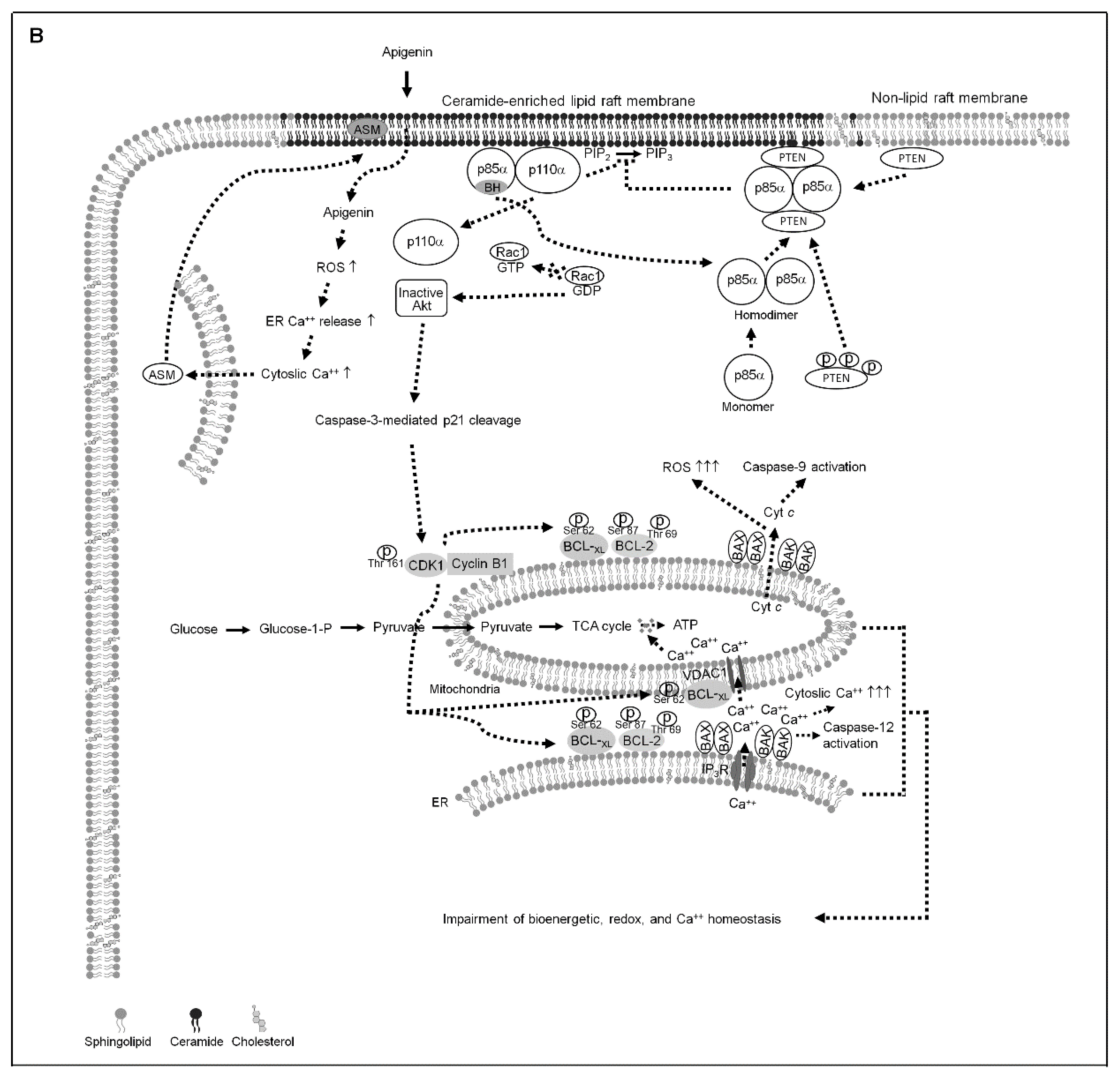
A molecular model for the attenuation of endoplasmic reticulum (ER)−mitochondria-mediated bioenergetic and lipid metabolic homeostasis by apigenin in nasopharyngeal carcinoma (NPC) cells. (**A**) The formation of clustered phosphatidylinositol 3-kinase (PI3K)−protein kinase B (Akt)−GTP-ras-related C3 botulinum toxin substrate 1 (Rac1) signaling platform in the lipid raft membranes constitutes a central element in the control of p21-mediated cyclin dependent kinase 1 (CDK1)−cyclin B1 inactivation to contribute initiation of B-cell lymphoma-extra large (BCL-x_L_)/ B cell lymphoma 2 (BCL-2)-inhibited Bcl-2-associated x protein (BAX)/Bcl-2 antagonist killer 1 (BAK) pro-apoptotic and metabolic activities, thereby modulates the ER−mitochondria-regulated bioenergetic, redox, and calcium (Ca^++^) homeostasis. (**B**) Under the condition of cellular apigenin uptake, apigenin induces the lipid raft membrane translocalization of acid sphingomyelinase (ASM) from lysosome to promote the generation of ceramide-enriched lipid raft membranes by triggering oxidative stress-induced ER Ca^++^ release, thus inducting the formation of p110α-free homodimerized p85α–unphosphorylated phosphatase and tensin homolog deleted from chromosome 10 (PTEN) tetrameric complexes and thereby disturbing the interaction between p85α and p110α in the lipid raft membranes. The resultant lipid raft membrane-associated p85α–PTEN complexes can negatively regulate Akt activity and attenuate Rac1 activation by dephosphorylating the 3-position of phosphatidylinositol-3,4,5-trisphosphate (PIP_3_) to phosphatidylinositol-4,5-bisphosphate (PIP_2_). Akt inactivation was accompanied by induction of caspase-3-mediated p21 cleavage resulting the mitochondrial localization of p-CDK1 (Thr 161)–cyclin B1 complexes, which causes the phosphorylation of ER–mitochondria-associated BCL-x_L_(Ser 62)/BCL-2 (Thr 69/Ser 87) and then results in the dimerization/oligomerization of BAX/BAK, thereby attenuating the regulatory effects of BCL-x_L_/BCL-2 on the control of ER−mitochondria-regulated bioenergetic, redox, and Ca^++^ homeostasis as well as resulting in apoptotic cell death of NPC cells.

Hyperactivation of PI3K−Akt signaling has been documented to confer survival advantage, metastatic potential, and resistance to chemotherapy in the NPC cells [59,60,61]. Accumulating evidence reveals that PI3K−Akt-mediated physiological communication between the mitochondria and ER is essential to maintain a proper transfer of Ca^++^ from the ER to the mitochondria required to regulate the control of mitochondrial bioenergetics and metabolism [62,63,64,65]. The important downstream effectors of PI3K−Akt pathway crucial for mediating cell survival are members of the BCL-2 family of proteins, BCL-2 and BCL-x_L_ [65]. Besides the anti-apoptotic function, BCL-2 and BCL-x_L_ are emerged as master regulators of ER and mitochondrial dynamics in cancer cells [6]. Upregulation of the anti-apoptotic action of BCL-2 and BCL-x_L_ was associated with apoptosis resistance of NPC cells [61,66]. It was therefore suggested that anti-NPC therapy can be achieved through disruption of PI3K−Akt-mediated oncogenic activities of BCL-2 and BCL-x_L_. The results of the present study are consistent with the previously demonstrated findings [12,20,67] that presence of BCL-2 (Thr 69/Ser 87) and BCL-x_L_ (Ser 62) phosphorylation mediated by mitochondria-localized CDK1–cyclin B1 activity results of the loss of their anti-apoptotic, bioenergetic, and metabolic functions of mitochondria. Modulation of mitochondrial oxidative metabolism fuel to produce ATP required for cancer cell survival was related to CDK1–cyclin B1 activity [20]. Inactivation of the PI3K−Akt signaling pathway by the PTEN through its potential binding with p110α-free p85α has been implicated in triggering the p-CDK1 (Thr 161)–cyclin B1 activity [29]. A p110α-free p85α–unphosphorylated PTEN complex formation in the lipid raft membranes is thought to the ability of p85α to active the lipid phosphatase activity of PTEN and thereby enhance its negative regulatory effect on the inactivation of Akt through dephosphorylating the 3-position of phosphatidylinositol-3,4,5-trisphosphate [54,68,69]. PTEN is referred to promote the apoptotic death of human pharyngeal squamous carcinoma cells by facilitating caspase-3-mediated p21 cleavage through an attenuation of Akt Ser 473 phosphorylation [29]. Its phosphoinositide phosphatase activity and lipid raft association has been shown to be induced by the lipid raft-localized ASM that efficiently induce ceramide-enriched lipid raft membranes, leading to the blocking of PI3K-mediated Akt (Ser 473) phosphorylation and activation [36]. Li et al. results indicate that Ca^++^-dependent membrane trafficking of ASM and ceramide-enriched membrane platforms caused by increased levels of ROS [57]. An appropriate level of the transfer of Ca^++^ from ER to mitochondria to regulate mitochondrial bioenergetics is recognized as important for the control of Ca^++^ and redox homeostasis [70,71]. During the induction of lipid raft-associated p110α-free p85α–unphosphorylated PTEN complexes by apigenin, ASM was recruited into the lipid raft membranes. Reduced Ser 473 phosphorylation and altered localization of Akt in the cytosol rather than the lipid raft membranes were observed in the apigenin-treated NPC cells. Full activation of Akt requires phosphorylation event of Ser 473 [72] and its location in the lipid rafts [73], and loss of Akt Ser 473 phosphorylation confers apoptotic death of NPC cells via dysfunction of ER and mitochondria functions [36]. Further study on the apigenin-induced inactivation of Akt by decrease of phosphorylation at Ser 473 correlates with the cleavage of p21 and pro-caspase-3. In view of the results of the present study observed elevated DRM ceramide level, lipid raft-associated p110α-free p85α–unphosphorylated PTEN complex formation, reduced the levels of DRM sphingomyelin and cholesterol, declined phosphorylation of Akt (Ser 473) and PTEN (Ser 380/Thr 382/Ser 385), aborted Rac activation, enhanced induction of p-CDK1 (Thr 161), p-BCL-x_L_ (Ser 62), p-BCL-2 (Thr 69/Ser 87), cytosolic Ca^++^ and ROS, decreased ATP level, and cell death after treatment of DOPC, an inducer of ASM activity in NPC cells. Treatment of cells with C2-ceramide resulted in effects that were similar to that of DOPC. The fact that an enhanced ceramide generation from hydrolysis of ASM-mediated sphingomyelin on the lipid raft membrane is sufficient to deplete the lipid raft membrane cholesterol [55,74]. The substitution of cholesterol by an increase amount of ceramide in the lipid rafts results in the lipid raft membrane targeting of PTEN and subsequently decrease in PI3K-mediated Akt activation [55,75,76]. In the present evidence that addition of ASM specific inhibitor imipramine or transfection of ASM shRNA causes increased levels of PI3K−Rac1−Akt activities and DRM cholesterol and decreased levels of Ca^++^ and ROS. Cells treating ASM shRNA or imipramine in the presence of apigenin exhibited rescue effects on the suppression of Akt (Ser 473) and PTEN (Ser 380/Thr 382/Ser 385) phosphorylation, Rac1 activation, p85α−p110α−GTP-Rac1 complex formation, DRM cholesterol and sphingomyelin levels, and cell growth and the induction of CDK1 (Thr 161) and BCL-x_L_ (Ser 62)/BCL-2 (Thr 69/Ser 87) phosphorylation, and as expected, PTEN was not co-immunoprecipitated with p85α. Cells depleted of ROS by a NAC were resistant to apigenin-induced the lipid raft membrane localization and activation of ASM, formation of ceramide-enriched lipid raft membranes and p110α-free p85α–unphosphorylated PTEN complexes, suppression of DRM cholesterol/sphingomyelin and ATP generation, and induction of BCL-x_L_(Ser 62)/BCL-2 (Thr 69/Ser 87) phosphorylation and ER−mitochondria-associated BAX/BAK oligomerization, and restored p85α−p110α−GTP-Rac1 complexes and ER–mitochondrial bioenergetic activities. The present data provide a novel role for oxidative stress-modulated Ca^++^-dependent localization of ASM into the lipid raft membranes in controlling the stabilization of PI3K–Rac1–Akt signaling platform that has physiological relevance related to allow the homeostasis of ER−mitochondria bioenergetic metabolism required for the progression of the NPC cell growth. The evidence from the present study, taken in conjunction with previous findings, we propose a working model where by apigenin can suppress the homeostasis of ER−mitochondria bioenergetic metabolism in NPC cells (Figure 7). These observations also illustrate PI3K−Rac1−Akt-modulated CDK1–cyclin B1–BCL-2/BCL-x_L_ pro-apoptotic activity and existence of considerable effect on the regulation of bioenergetic and metabolic functions.

The potential function of BCL-2 and BCL-x_L_ is initially characterized to regulate the mitochondrial membrane permeability, the role of BCL-2 and BCL-x_L_ on the modulation of ER functions is received increasing evidence [77]. Despite their biological relevance is highly conserved molecular process of anti-apoptotic action involved in the regulation of cells with DNA-damage responses [78]. BCL-x_L_ preferentially exerted mitochondrial anti-apoptotic and mitochondria-protective effects by interacting with mitochondrial voltage-dependent anion channel 1 (VDAC1) to modulate mitochondrial Ca^++^ uptake [79,80], it also acts for the balance of ER Ca^++^ level to decline cytosolic Ca^++^ by inhibiting the pro-apoptotic activity of BAX/BAK [81]. In contrast, BCL-2 plays an essential mediator of Ca^++^ trafficking from ER to mitochondria and cytosol by interacting with inositol 1,4,5-trisphosphate receptor, thus modulates ER Ca^++^ release and prevents apoptosis action of BAX/BAK [82,83,84]. Moreover, BCL-2 is still able to exert its anti-apoptotic activity suppressing mitochondrial apoptotic pathway [78]. The importance of BCL-x_L_ over BCL-2 regarding the regulation of mitochondrial function in NPC cells is thought to come the ability of its overexpression to overcome pro-apoptotic signal from the inactivation of PI3K−Akt pathway [37]. The results presented here show that BCL-x_L_ appears to play a prominent role in regulation of the generation of mitochondrial ATP and ROS, and contributes to the canonical role in preventing of the oligomerization of ER/mitochondria-associated BAX/BAK. In addition to antagonize BAX/BAK oligomerization, BCL-2 is most important for the regulation of the release of Ca^++^ from the ER to cytosol. Considering these findings and the observed presence of p-BCL-x_L_(Ser 62) and p-BCL-2 (Thr 69/Ser 87) causing the oligomerization of BAX/BAK at the ER/mitochondria and the dysregulation of bioenergetic metabolism after treatment with apigenin, it is logical to suggest that the phosphorylation status of BCL-x_L_(Ser 62) and BCL-2 (Thr 69/Ser 87) at the ER and mitochondria has physiological relevance related to the modulation of BAX/BAK pro-apoptotic activity to decide the process of ER–mitochondria-regulated energy synthesis required for NPC cell growth.

In most types of human cancer cells, active PI3K–Akt signaling acts as a crucial coordinator of glycolytic and lipid metabolism to conduct the metabolic reprogramming of cancer cells by regulating the glucose transporter-1 (GLUT-1)-mediated glucose uptake [85,86]. Recent studies indicate that a declined Akt activity involving in the low rate of glucose uptake to decrease ATP synthesis of NPC cells was achieved through downregulating of GLUT-1 to the lipid raft membranes [38]. By using shRNA targeting and pharmacological inhibition strategies in which the oxidative stress-induced ASM activity was attenuated, we have demonstrated here that formation of ceramide-enriched lipid raft domains caused perturbation of p110α, Akt, and Rac1 lipid raft compartmentalization and resulted in the loss of their activity. Changing the component properties of cholesterol and sphingolipid-enriched lipid rafts disorganized the localization of signaling effectors or receptors to the lipid raft membranes [75]. Cells expressing FLAG-BCL-x_L_ (S62A) or BCL-2 (T69A/S87A) did not display a significant suppression in the decrease of cellular glucose level by apigenin. CDK1 siRNA or cyclin B1 siRNA expression, or Ro-3308 co-treatment was slightly but significantly inhibited the apigenin-induced decline of cellular glucose. Based on these observation, we speculated that formation of oxidative stress-induced ceramide-enriched lipid raft membranes caused by apigenin may result in delocalization of GLUT-1 from the lipid raft membranes, leading to a decrease in glucose uptake.

The present study’s results represent an important advance in our understanding of the regulation of ER−mitochondria-modulated bioenergetic metabolism in NPC cells. These findings not only have potential implications for resolving the molecular mechanism of apigenin suppression of NPC cell growth, but also provide a theoretical basis for the further development of novel oxidative stress or ASM inducers.

## 5. Conclusions

This is the first finding that suggests apigenin-triggered oxidative stress initiates the lipid raft membrane localization of Ca^++^-dependent ASM forming a ceramide-enriched lipid raft membrane platform, leading to mislocalization of PTEN into the lipid rafts to form complex with p85α, thereby attenuating the PI3K−Rac1−Akt-mediated signaling. Akt inactivation causes the mitochondrial localization of p-CDK1 (Thr 161)–cyclin B1 complexes. The resultant mitochondria-associated p-CDK1 (Thr 161)–cyclin B1 complexes triggers phosphorylation of BCL-x_L_(Ser 62) and BCL-2 (Thr 69/Ser 87), resulting in the loss of BCL-x_L_/BCL-2-dictated ER–mitochondria bioenergetic metabolism. Thus, the molecular mechanism underlying apigenin-induced inhibition of NPC cell growth may reveal strategy for theoretical basis of new concept in anticancer treatment.

## Data Availability

All results generated or analyzed during present study are included in this published article. Data and materials will be made available upon request via email to corresponding author (dr.chen3693@gmail.com).

## Abbreviations

Acid sphingomyelinase	ASM
B cell lymphoma 2	BCL-2
Bcl-2-antagonist of cell death	BAD
Bcl-2 antagonist killer 1	BAK
Bcl-2-associated x protein	BAX
BCL-2/B-cell lymphoma-extra large	BCL-x_L_
Cyclin dependent kinase 1	CDK1
Cytochrome *c*	Cyt *c*
Endoplasmic reticulum	ER
Phosphatase and tensin homolog deleted from chromosome 10	PTEN
Phosphatidylinositol 3-kinase	PI3K
Phosphatidylinositol-4,5-bisphosphate	PIP_2_
Phosphatidylinositol-3,4,5-trisphosphate	PIP_3_
Poly (ADP-ribose) polymerase	PARP
Protein kinase B	Akt
Ras-related C3 botulinum toxin substrate 1	Rac1
Short hairpin RNA	shRNA
Small interfering RNA	siRNA
Nasopharyngeal carcinoma	NPC
